# RPE and neuronal differentiation of allotransplantated porcine ciliary epithelium-derived cells

**Published:** 2011-10-05

**Authors:** Jasenka Guduric-Fuchs, Wing Chen, Henrietta Price, Desmond B. Archer, Tiziana Cogliati

**Affiliations:** 1 Centre for Vision and Vascular Sciences, Queen’s University Belfast, Royal Victoria Hospital, Institute of Clinical Science, Northern Ireland, UK; 2 Royal Victoria Hospital, Grosvenor Road, Belfast, Northern Ireland, UK; 3 The Agri-Food and Biosciences Institute (AFBI), Chemical Surveillance Branch, Veterinary Sciences Division, Stoney Road, Stormont, Belfast, Northern Ireland UK

## Abstract

**Purpose:**

Cell replacement has the potential to be applied as a therapeutic strategy in retinal degenerative diseases such as retinitis pigmentosa and age-related macular degeneration (AMD) for which no adequate pharmacological and surgical treatments are currently available. Although controversial, the use of ciliary epithelium (CE)-derived cells is supported by evidence showing their differentiation into retinal phenotypes. This study examines the differentiation potential of porcine CE-derived cells in vitro and their survival, migration, morphological characteristics, and immunohistochemical phenotype in vivo, upon transplantation into the subretinal space of normal pigs.

**Methods:**

Cells were isolated from the CE of postnatal pigs and were grown in a suspension sphere culture. Differentiation was assessed in vitro after exposure to laminin and the addition of serum. For transplantation, CE-derived spheres were dissociated, labeled with CM-DiI vital dye, and the cells were injected subretinally into one eye of eight week-old allorecipients. The eyes were examined at eight days and at two and four weeks after transplantation.

**Results:**

Cells positive for neuronal and retinal pigment epithelium (RPE) markers were detected by immunohistochemistry in differentiation cultures. Reverse Transcriptase-Polymerase Chain Reaction (RT–PCR) revealed upregulation of neuronal markers after in vitro differentiation. CM-DiI dye-labeled CE-derived cells dissociated from primary spheres survived for up to four weeks after transplantation in vivo. Some of the surviving cells migrated distantly from the injection site. Large clusters of transplanted cells integrated into the RPE layer and multilayered RPE-like structures positive for RPE65 were often observed. Grafted cells were also identified in the neuroretina where 5%–10% were positive for recoverin, protein kinase C alpha (PKCα), and calbindin.

**Conclusions:**

The efficient conversion to an RPE-like phenotype suggests that CE-derived cells could be a potential source of RPE for cell replacement. Our data also suggest that the ability of these cells to acquire neuronal phenotypes is influenced by the environment. Thus, pre-differentiated or (re)programmed CE-derived cells may be more amenable for retinal repair.

## Introduction

Cell replacement is a promising approach to restore neural function in the degenerating nervous system, including the retina. Since retinal dystrophies are ultimately characterized by the loss of photoreceptors, efforts have been made in the last decade to identify suitable sources of stem/progenitor cells and drive their differentiation along the photoreceptor lineage in vitro and in vivo. Several cell populations with retinal progenitor properties have been identified in the eye, including Müller glia, ciliary epithelium (CE)-derived, and iris-derived, and their ability to generate retinal cell types has been reported [1-4]. CE-derived cells have been shown to display stem/progenitor cell features, including clonal expansion and differentiation toward retinal phenotypes under appropriate conditions in vitro and in vivo [5-8]. The CE is located in a surgically accessible part of the eye, therefore, cells derived from this tissue offer an attractive possibility for autologous transplantation.

It is well established that continuous growth of the eye in lower vertebrates such as fish and frogs depends on the retinal stem cells located in the ciliary marginal zone [9]. A similar but less potent stem cell zone has also been identified in chickens [10]. Although an analogous structure does not exist in mammals, it has been proposed that multipotent retinal stem cells can be isolated from the CE [5,6]. However, the nature and the developmental potential of cells derived from the CE have been the object of controversy in recent literature. First, the existence of a small quiescent population of retinal stem cells (RSCs) in the CE that can be propagated in vitro has been challenged [11]. Second, the “stemness” of cells in neurospheres derived from the CE has been questioned on the basis of the persistence of pigmentation and of their expression of makers and the characteristics of pigmented CE [11]. Finally, doubts have been expressed about the developmental potential of CE-derived cultures and their capacity to differentiate along retinal lineages [11,12]. While all published literature is concordant in reporting the limited self-renewal capacity of CE-derived cells [5,7,8,13,14], work from several laboratories has shown their differentiation, both in vitro and in vivo, into neuronal and photoreceptor-like phenotypes [7,13,15,16]. Thus, although the definition as RSCs might not be the most appropriate, further investigation is needed to test the potential of CE-derived cells to generate retinal photoreceptors either by direct differentiation, transdifferentiation, or genetic manipulation.

Due to its close similarity to the human eye, a pig eye provides an appropriate system for the evaluation of potential therapeutic strategies for retinal degeneration [17,18]. Furthermore, the size of a pig eye enables accurate dissection of the ciliary epithelium without contamination from tissues such as the retina or RPE. In addition, pig eyes can be freshly harvested from euthanized animals, offering an advantage over human specimens that are usually available for research after a prolonged post-mortem period. Although porcine CE-derived cells have been isolated and studied before by the authors of this report and by others [8,19], this is the first study to include the subretinal transplantation of these cells. To date, the transplantation studies using CE derived cells have only been performed in murine animal models, although xenotransplantation of human cells into a developing mouse eye has also been performed [7,11,20]. Here, we evaluated the ability of postnatal porcine CE-derived cells to generate retinal cell types in vitro and when injected subretinally into allorecipient eyes. We adopted surgical procedures similar to those used for subretinal transplantation of fetal retinal progenitor cells in pigs [21,22].

## Methods

All animals procedures were approved by The Queen’s University of Belfast Animal Ethics Committee and were performed in accordance with the UK Animals (Scientific Procedures) Act, 1986 and the ARVO statement on animal use. Mixed sex, white Landrance pigs were obtained from Agri-Food and Biosciences Institute (Hillsborough, Northern Ireland).

### Cell isolation and culture

One to two week-old piglets were anaesthetized with 15 mg/kg of intra-muscular azaperone (Stresnil, Janssen Animal Health, Saunderton, UK) and 20 mg/kg ketamine (Ketaset, Fort Dodge Animal Health, Southampton, UK) and were euthanized by intravenous or intra-cardiac injection of 100 mg/kg pentobarbitone (Pentoject, Animalcare, Masham Ripon, UK). The eyes were enucleated and placed into oxygenated artificial cerebral spinal fluid (aCSF: 124 mM NaCl, 5 mM KCl, 1.3 mM MgCl_2_, 26 mM NaHCO_3_, and 10 mM D-glucose, pH 7.5). The eyes were bisected at the ora serrata. The vitreous was decanted from the anterior half and the lens was removed. The ciliary body was dissected from the iris and pars plana. The strips of ciliary body were enzymatically digested in Hanks’ Balanced Salt Solution (HBSS) containing 2 mg/ml dispase (all from Sigma-Aldrich, Poole, UK) for 20 min at 37 °C, followed by digestion in Earle's Balanced Salt Solution (EBSS) containing 1.33 mg/ml trypsin, 0.67 mg/ml hyaluronidase, and 78 units/ml collagenase (Sigma-Aldrich) for 20 min at 37 °C. The supernatant was decanted and replaced with a serum-free medium (SFM, DMEM/F12 [1:1] containing 0.6% [w/v] glucose, 2 mM glutamine, 5 mM HEPES buffer, 2% [v/v] B27, 100 units/ml penicillin, and 100 units/ml streptomycin) with 1 mg/ml trypsin inhibitor (Invitrogen, Paisley, UK) and was incubated for 5 min at room temperature. The strips of ciliary body were subsequently placed in a 60 mm cell culture dish containing the SFM. Epithelial cells were peeled off and the non-epithelial tissue was discarded. The epithelial cellular debris was gently triturated 10–15 times using a pipette. Cells were pelleted at 1,000× g for 10 min, resuspended in the SFM, and were passed through a 40 μm cell strainer (BD Biosciences, Franklin Lakes, NJ). The cells were counted and plated at a density of 3×10^4^ cells/ml in the SFM supplemented with 20 ng/ml of an epidermal growth factor (EGF, Invitrogen) and 20 ng/ml of a basic fibroblast growth factor (bFGF, Invitrogen). After seven days, newly formed sphere colonies were collected, pelleted at 1,000× g for 10 min, digested in an Accumax cell counting solution (ICT, San Diego, CA) for 20 min at 20 °C, and were mechanically dissociated into single cells by pipetting and replating at a density of 3×10^4^ cells/ml.

For differentiation, CE-derived spheres were collected at the first passage, plated on poly-D-lysine and laminin-coated glass coverslips (BD Biosciences), and were allowed to differentiate for 20 days in the presence of either fetal calf serum (1%, 5%, and 10%), or of 1% serum with growth factors (10 ng/ml of EGF and bFGF). The medium was replaced every three days. After 20 days of differentiation, the cells were fixed in 4% PFA for 20 min at room temperature and were processed for immunocytochemistry.

For cell transplantation, spheres from the first passage were collected and dissociated into single cells using Accumax (ICT, San Diego, CA). The cells were labeled with CM-DiI (Invitrogen) following the manufacturer's protocol and were injected as described below.

### Conventional RT–PCR

Total RNA was extracted using an RNeasy Mini Kit (Qiagen, Crawley, UK). On column DNaseI digestion was performed to digest any contaminating genomic DNA. One µg of RNA was reverse transcribed using random primers and SuperScript II (Invitrogen) according to the manufacturer’s instructions. No RT controls were performed by omission of reverse transcriptase in the reaction. PCR was performed in a 30 μl reaction volume containing 1 μl of cDNA, 0.2 μM sense and anti-sense primers, 1× PCR buffer (Qiagen), 10 mM dNTP mix (Roche, Burgess Hill, UK), and 1 μl Hot Start DNA polymerase (Qiagen). Primer sequences are shown in Table 1. PCR was performed for 40 cycles using a thermocycler (ABI 2720, Applied Biosystems, Foster City, CA). PCR products were resolved on 1.5% agarose gel.

**Table 1:** Primer sequences used for RT–PCR.

**Gene**	**Accession No (reference)**	**Forward primer**	**Reverse primer**	**Product size (bp)**
*Nanog*	NM_001129971.1	TGGAGTAACCCAACCTGGAG	ATGATTTGCTGCTGGGTACC	269
*0ct4*	NM_001113060.1	GTTTTGAGGCTTTGCAGCTC	TCTCCAGGTTGCCTCTCACT	183
*cMyc*	NM_001005154.1	GGAAGGACTATCCCTCTGCC	TCCAACTCTGGGATCTGGTC	208
*Klf4*	NM_001031782.1	CAGCTTCAGCTATCCGATCC	TGATGTCTGCCAGGTTGAAG	128
*Sox2*	[21]	GGCAGCTACAGCATGATGCAGGAGC	CTGGTCATGGAGTTGTACTGCAGG	131
*Six3*	[21]	AGCGGACTCGGAGCCTGTTG	AGCGCATGCCGCTCGGTCCA	202
*Otx2*	XM_003121824.1	GCTGTGTGAATTGTGCGACT	GGTGGAGTTCAAGGTTGCAT	193
*Mitf*	NM_001038001.1	GGGCCGCCTAAAGCGTGGT	GGTCGCCAGGCTGGTTTGGAC	198
*Chx10*	NM_182894.2	AGGGAGAACAGCATTGCGGTGC	GCGCCTTGACCTAAGCCATGTCC	193
*Hes1*	NM_001195231.1	CAGCCAGTGTCAACACGACAC	TCGTTCATGCACTCACTGA	307
*HPRT*	NM_001032376.2	CCAGTCAACGGGCGATATAA	CTTGACCAAGGAAAGCAAGG	130
PKC alpha	XM_003131278.1	GACCATCCGCTCTACACTCAAC	CCCAGTCCCAGATTTCTACAG	104
Calbindin	NM_001130226.1	TCTGCTGGGGACAACTAAATTT	CAGCCTACTCCGTTACAGTGCA	93
Rhodopsin	NM_214221.1	TCCATCTACAACCCCGTCAT	CTGTCTTGGAAGTGGTGGTG	127

Primers were either designed from sequences retrieved under the accession numbers shown, or taken from published studies.

### Real time RT–PCR

For differentiation, CE-derived spheres were collected at the first passage, plated on poly-D-lysine and laminin-coated six well plates (BD Biosciences), and were allowed to differentiate for 20 days in the presence of 1% serum and 10 ng/ml of EGF and bFGF. The medium was replaced every three days. After 20 days, the cells were harvested and RNA was isolated and reverse transcribed as outlined above. Real time PCR was performed with 2× Maxima SYBR Green qPCR Mastermix (Fermentas, Cambridge, UK) in 10 µl reactions containing 2 µl of 1:15 cDNA dilution and 0.5 µM of the gene specific primer. Primer efficiencies were determined from standard curves constructed using serial dilutions of pooled cDNA. Hypoxathineposphoribosyltransferase (*HPRT*) was used as the housekeeping gene for normalization. Primer sequences are shown in Table 1. Reactions were performed on a LightCycler PCR system (Roche) with the following program: initial denaturation at 95 °C for 10 min, followed by 40 cycles at 95 °C for 15 s, 58 °C for 10 s, and 72 °C for 15 s. Relative gene expression (including statistical analysis) was determined using REST software. The RNA from three independent experiments was analyzed and all reactions were performed in triplicate.

### Surgical procedure

One eye from eight week-old (weight from 17.5 to 21 kg) female pigs (n=8) was transplanted. Prior to transfer to the operating theater, the animals were sedated with 2 mg/kg of azaperone (Stresnil; Janssen Animal Health, Saunderton, UK) by intramuscular injection. In the theater, the animals were sedated by intramuscular injection of 1 mg/kg xylazine (Rompun 2%; Bayer, Newbury, UK) and 4 mg/kg of ketamine hydrochloride (Ketaset 100 mg/ml; Fort Dodge, Southampton, UK), followed by administration of 0.2 mg/kg morphine (Morphine sulfate 10mg/ml; controlled drug [CD], UK). Anesthesia was induced for tracheal intubation with 1 mg/kg intravenous alfaxalone (Alfaxan 10mg/ml; Vetoquinol, Buckingham, UK), and was maintained using 1%–1.75% isoflurane in oxygen. Approximately 0.5 l of lactated Ringer’s (Hartman’s) isotonic solution was infused intravenously during anesthesia.

The pupil in each eye was dilated with topical medication (1 to 3 drops each of Gt cyclopentolate 1% and Gt phenylephrine 2.5%). A standard three-port pars plana vitrectomy was performed. The sclerotomies were positioned 2 mm posterior to the limbus. A retinotomy in the area centralis was performed using a 42G needle (Bausch and Lomb, Whelehan Group, Dublin). A small subretinal air bubble was created through the retinotomy, followed by the injection of 1x10^6^ cells in a maximum volume of 0.1 ml of phosphate-buffered saline (PBS) into the subretinal space. The sclerotomies were closed using 7/0 braided polyglactin sutures (Vicryl; Ethicon, Livingston, UK).

One pig was killed before recovery from anesthesia by intravenous pentobarbitone overdose at the end of the surgery. An intravenous injection of 2–4 mg/kg carprofen (Rimadyl Large Animal 50 mg/ml; Pfizer, Sandwich, UK) was administered to the other pigs after transplantation and before recovery from the anesthetic (by discontinuation of isoflurane), and xylazine sedation was reversed as necessary using approximately 0.2 mg/kg atipamezole (Antisedan 5 mg/ml; Pfizer). The animals were kept in a warm chamber for the first day after surgery. Topical eye drops containing 0.3% tobramycin and 0.1% dexamethasone (Tobradex; Alcon, Hemel Hempstead, UK) were instilled at the end of surgery, and then daily for 14 days post surgery.

At 8, 14, or 28 days after transplantation the animals were lethally anaesthetized with intravenous injection of pentobarbitone (2–4 g), and their eyes were enucleated and washed in PBS. After removal of the cornea and lens, the eyes were fixed in 4% PFA in PBS for 1 h at room temperature. The eyecups were cryoprotected in 10% sucrose for 6 h followed by 30% sucrose overnight, embedded in an optimal cutting temperature (OCT) compound (Sakura, Kobe, Japan), and were snap frozen in an isopentane bath on dry ice. Transverse cryosections (20 µm) were cut, mounted onto Superfrost Plus glass slides (Fisher Scientific, Loughborough, UK), and stored at −80 °C until used.

### Immunohistochemistry

Immunohistochemistry on tissue sections was performed as described previously [18]. Briefly, slides were thawed at room temperature and were post-fixed in 4% formaldehyde (Sigma-Aldrich) in PBS for 20 min at room temperature. After rinsing in PBS, sections were blocked for 1 h in 10% normal goat serum (NGS), 0.3% Triton X-100, 0.01% NaN_3_ in PBS, at room temperature. Slides were incubated for 24 h at 4 °C with a primary antibody diluted in 10% NGS, 0.3% Triton X-100, and 0.01% NaN_3_ in PBS. The primary antibodies used are listed in Table 2. After removal of the primary antibody, slides were washed 6×5 min in PBS and were incubated for 1 h at room temperature in a secondary antibody (Alexa Fluor^488^ goat anti-mouse or goat anti-rabbit), 1:500 in PBS. After 3×5 min washing steps in PBS, cell nuclei were counterstained with 5 μM DAPI (Invitrogen) for 10 min. The slides were mounted in a fluorescent mounting medium (Dako, Ely, UK). Negative immunohistochemistry controls were performed in parallel by omission of the primary antibody. Immunoreactive cells were visualized and images were recorded using an inverted confocal microscope (Nikon, Model Eclipse TE 2000-U, Tokyo, Japan) and Nikon EZ-C1 software. Every tenth or twentieth section (200–400 µm step) was stained for the same antibody.

**Table 2:** Primary antibodies used for immunohistochemical analysis.

**Antibody**	**Host**	**Dilution**	**Source**
Recoverin	rabbit	1:1000	Kind gift from Karl-Wilhelm Koch
Rhodopsin (Rho4D2)	mouse	1:100	Kind gift from Robert Molday
PKCα	mouse	1:400	Sigma-Aldrich
Calbindin	rabbit	1:1500	Chemicon, Millipore
RPE65	mouse	1:400	Chemicon, Millipore
Ki67	mouse	1:300	BD Biosciences
Neurofilament (NF)-M	mouse	1:350	Sigma-Aldrich
HuC/D	mouse	1:200	Molecular probes, Invitrogen

Host animal, dilution and source for each antibody are shown.

For isolectin B4 staining, sections were blocked in 5% BSA for 30 min, incubated with biothynilated Griffonia simplicifolia Isolectin B4 (Vector) 1:100 for 1 h, washed for 3×5 min with PBS, and were finally incubated with streptavidin-FITC 1:200 for 1 h.

For the immunocytochemistry of the differentiated cells, post-fixation glass slides were washed 3× in PBS, incubated in 10% NGS, 0.3% Triton X-100, and 0.01% NaN_3_ in PBS for 1 h at room temperature, followed by overnight incubation at 4 °C. The slides were incubated for 24 h at 4 °C with the primary antibody diluted in 10% NGS, 0.3% Triton X-100, and 0.01% NaN_3_ in PBS. For double labeling, the second primary antibody was added after removal of the first primary antibody and was incubated for another 24 h at 4 °C. After removal of the second primary antibody, the slides were washed for 6×5 min with PBS and were incubated in the first secondary antibody (Alexa Fluor488 goat antimouse) diluted 1:500 in PBS for 1 h. Subsequently, incubation with another secondary antibody (Alexa Fluor568 goat antirabbit), was performed for 1 h. The slides were washed for 3×5 min with PBS and were counterstained and mounted as described above. The cells were visualized and the images were captured with an epifluorescence microscope (Nikon) using Nis Elements (Nikon) software. The number of positive cells was counted in 20 random fields at 40× magnification.

## Results

### Analysis of the gene expression of CE-derived spheres

**Figure 1:**
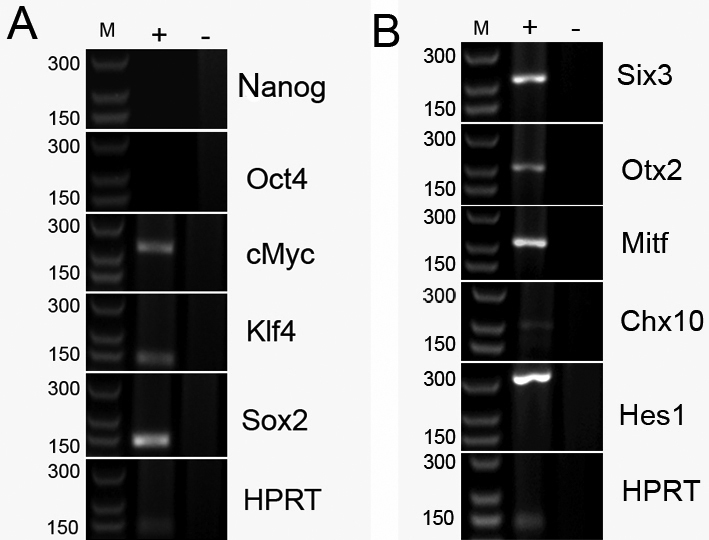
Gene expression of ciliary epithelium (CE)-derived cells determined by RT–PCR. RNA was isolated from passage 1 and was subjected to conventional RT–PCR. PCR products were resolved on 1.5% agarose gel. **A**: Amplification of mRNA for pluripotency markers. **B**: Amplification of mRNA for retinal progenitor genes. Sizes in base pairs for the corresponding marker bands (M) are shown on the left, adjacent to the gel images. PCR reactions performed with cDNA template are shown in lanes marked as '+' and negative control reactions performed with templates from the RT where reverse transcriptase was omitted are shown in lanes marked as '–'. Amplicons for a housekeeping gene (HPRT) under the same conditions are shown in the bottom panels.

Expression of the key pluripotency genes [23,24] and the genes active during normal retinal development was analyzed by RT–PCR using RNA extracted from P1 CE-derived spheres. Transcripts for three pluripotency genes, namely *cMyc*, *Klf4*, and *Sox2* were present in CE-derived cultures, while mRNAs for *Nanog* and *Oct4* were not detected (Figure 1A). Transcription factors associated with the eye specification and retinal histogenesis, including Six3, Mitf, Hes1, Otx2 and Chx10, were also expressed in CE-derived spheres (Figure 1B).

### In vitro differentiation of CE-derived spheres

**Figure 2:**
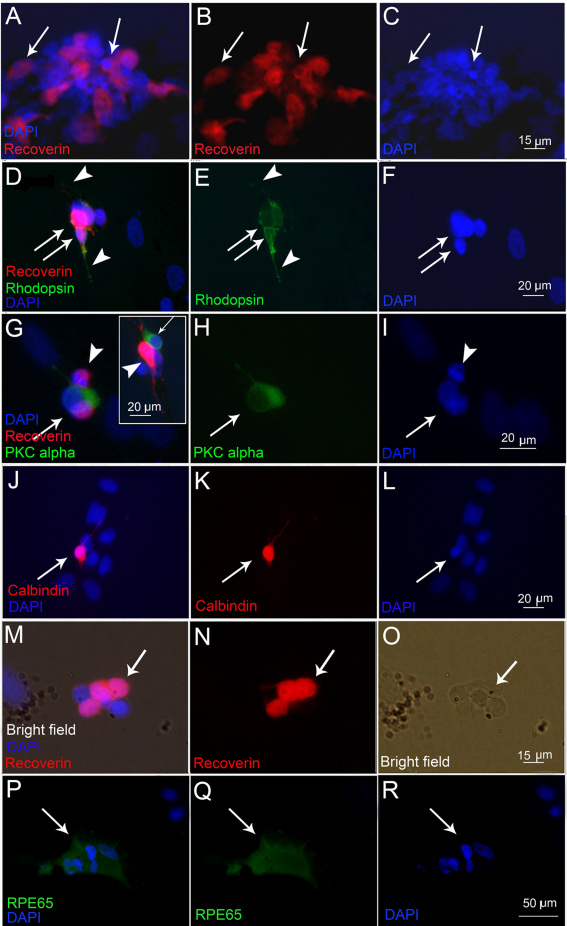
Microphotographs of the immunolabeling of newborn pig ciliary epithelium (CE)-derived cells after in vitro differentiation on poly-D-Lysine, laminin coated coverslips in the presence of 1% serum and 10 ng/ml basic fibroblast growth factor (bFGF) and epidermal growth factor (EGF). The images were acquired using an epifluorescent microscope. **A**, **B**: Cells labeled for recoverin are clustered together (arrows). **C**: 4',6-diamidino-2-phenylindole (DAPI) nuclear staining corresponding to **A** and **B**. **D**, **E**: Cells double-labeled for recoverin (red, **D**) and rhodopsin (green, **D** and **E**) are depicted by arrows. The focus is set to show rhodopsin-positive cell processes. Strong recoverin staining in the cytoplasm masks the nuclear DAPI staining, which is shown separately in **F**. Cells positive for protein kinase α (PKCα; green in **G** and **H**, arrows) did not co-label with recoverin (red in **G** and **H** and another example in the inset in **G**, arrowheads). The focus is set to show the processes of PKCα-labeled cells in **G** and **H**, and the recoverin-labeled processes in the inset in **G**. Corresponding DAPI nuclear stain is shown in **I**. **J**-**K**: A calbindin immunopositive cell is depicted by the arrow. The focus is set to show the processes of the labeled cell. Corresponding DAPI nuclear stain is shown in **L**. **M**, **N**: Recoverin-positive cells (arrows in **M** and **N**) that had retained pigmented granules (arrow in the bright-field image in **O**). **P**, **Q**: RPE-65 immunopositive cells (arrows) and the corresponding nuclear DAPI staining in **R**.

The capacity of CE-derived cells from newborn pigs to differentiate into retinal phenotypes was first evaluated in vitro, after plating CE spheres on adherent substrates (poly-D-lysine and laminin) and culturing for 20 days with a differentiation medium containing serum and growth factors. Growth factors (10 ng/ml bFGF and EGF) enhanced retinal differentiation in the presence of 1% serum (Figure 2). Photoreceptor markers recoverin (Figure 2A-C) and rhodopsin (Figure 2D-F), the bipolar cell marker PKCα (Figure 2G-I), the ganglion, amacrine, and horizontal cell marker calbindin (Figure 2J-L) and the RPE marker RPE65 (Figure 2P-R) were detected by immunocytochemistry in different proportions of cells. Labeling specificity was verified on mouse skin fibroblasts as negative controls and on pig retinal progenitor cells as positive controls (data not shown). Recoverin labeling was detected in about 20±3.2% of cells, rhodopsin labeling in 14.5±3.2%, PKCα labeling in 19.3±4.1%, and calbindin labeling in 21.4±2.4% of cells. Cells immunopositive for neuronal markers in vitro extended thin, long processes, which are suggestive of neuronal differentiation. Rhodopsin-labeled cells were positive for recoverin in double labeling experiments (Figure 2D,E). Double labeling also revealed that PKCα and recoverin antibodies stained a distinct population of cells and there was no overlap between these two markers. However, PKCα-labeled cells were usually found adjacent to recoverin-positive cells (Figure 2G). RPE65 immunoreactivity was detected in 12.2±3.8% cells. Cells within the spheres remained pigmented and although rare, pigment granules were sometimes observed within the cells expressing retinal markers (Figure 2M-O).

**Figure 3:**
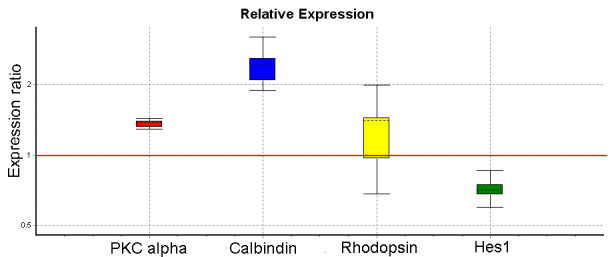
Quantitative real time PCR data for ciliary epithelium (CE)-derived cell cultures following in vitro differentiation. RNA was isolated from CE-derived cells after in vitro differentiation on poly-D-Lysine, laminin coated plates in the presence of 1% serum and 10 ng/ml basic fibroblast growth factor (bFGF) and epidermal growth factor (EGF) for 20 days. The data was analyzed using REST software for the relative quantification. The expression ratio represents the ratio of expression in differentiated compared to undifferentiated cultures. After differentiation, protein kinase α (PKCα; p<0.05), calbindin (p<0.05), and rhodopsin (p=0.054) were upregulated, while the retinal progenitor marker Hes1 was downregulated (p<0.01). The data are from a representative experiment performed in triplicate. Similar results were obtained from three independent differentiation experiments.

Real time PCR confirmed upregulation of PKCα (p<0.05), calbindin (p<0.05), and rhodopsin (p=0.053) in differentiation cultures, relative to their expression level before differentiation. Concomitantly, the retinal progenitor marker Hes1 was downregulated after differentiation (Figure 3).

### Transplantation of CE-derived cells

**Figure 4:**
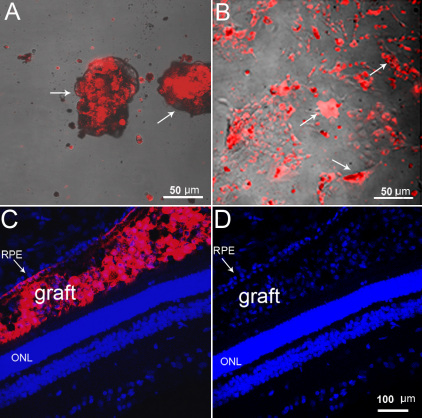
CM-DiI labeling of ciliary epithelium (CE)-derived cells. Dissociated cells at passage 1 were labeled and cultured in a suspension culture to form spheres (**A**), or were plated on poly-D-Lysine, laminin coated coverslips in differentiation conditions. The CM-DiI label was retained for up to 10 days in proliferating spheres (depicted by arrows in **A**), and up to four weeks in differentiating conditions (arrows in **B**) in vitro. **C**, **D**: Microphotographs of grafted CM-DiI-labeled CE-derived cells (red) in the recipient retina 10 min after subretinal injection. **C**: CM-DiI labeling merged with nuclear DAPI staining. **D**: 4',6-diamidino-2-phenylindole (DAPI) staining only. Red CM-DiI-labeled cells were found between the RPE (arrow) and the ONL. Retinal pigment epithelium (RPE); outer nuclear layer (ONL).

Prior to initiating in vivo transplantation experiments, the CM-DiI dye was tested for long-term stability. CM-DiI showed long-term retention in CE-derived cells in proliferating (10 day follow-up) and differentiating (4 week follow-up) conditions in vitro (Figure 4A-B). In vivo, subretinal localization of CM-DiI-labeled cells 10 min after grafting was confirmed in cryosections from one animal. CM-DiI-labeled cells (red) were found between the RPE and outer nuclear layer (ONL). Retinal detachment at the injection area was also observed (Figure 4C,D).

### Incorporation of transplanted cells into the RPE layer and formation of multilayered RPE-like structures

**Figure 5:**
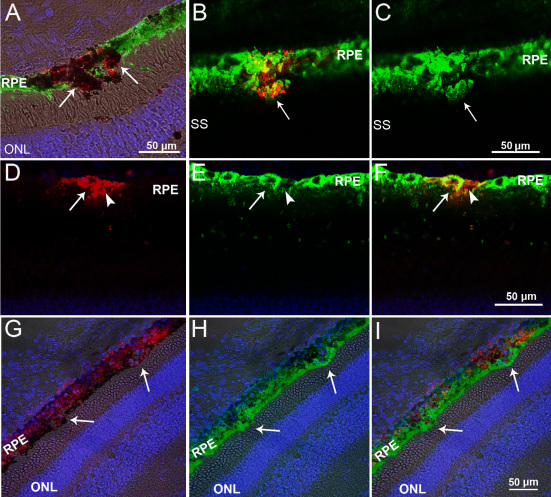
Microphotographs of red CM-DiI-labeled ciliary epithelium (CE)-derived cells in the retinal pigment epithelium (RPE) layer. **A**: Red-labeled pigmented CE-derived cells localized to the RPE layer, which were negative for RPE65 (arrows). **B**: At the same time point, transplanted red-labeled RPE65-positive cells were also found (red and green merged in **B** and green RPE65 labeling only in **C**, arrows). **D**-**F**: Two weeks following transplantation, CM-DiI-labeled cells in the RPE layer were strongly (arrow) and weakly (arrowhead) positive for RPE65. **G**-**I**: Four weeks after transplantation, the RPE appeared uneven and multilayered (arrows). Nuclei are labeled with 4',6-diamidino-2-phenylindole (DAPI; blue). Bright-field images are merged with the dark field in **A**, **G**, **H,** and **I.** Subretinal space (SS); and outer nuclear layer (ONL).

Eight days following transplantation, large CM-DiI positive cell aggregates were observed within the RPE layer (Figure 5). Clusters of CE-derived cells in the RPE were either RPE65-negative (Figure 5A), or showed strong RPE65 immunoreactivity (Figure 5B,C). At two and four weeks after transplantation, many CM-DiI-labeled cells were localized in the RPE layer (Figure 5D-I). Due to the phagocytic nature of the RPE, some of the CM-DiI labeling in this layer may be attributed to the uptake of the dye from dead transplanted cells. However, four weeks following transplantation, areas of the RPE were often multilayered, suggesting the de novo formation of additional RPE-like layers on top of the host RPE on the basal side (Figure 5G-I). The thickness of the RPE increased due to the formation of multilayers; in some areas it was comparable to that of the ONL (Figure 5G-I). Some CM-DiI-labeled cells were also observed at the level of the choroid, but they were negative for RPE65.

### Migration of transplanted cells into the neuroretina and expression of retinal markers

**Figure 6:**
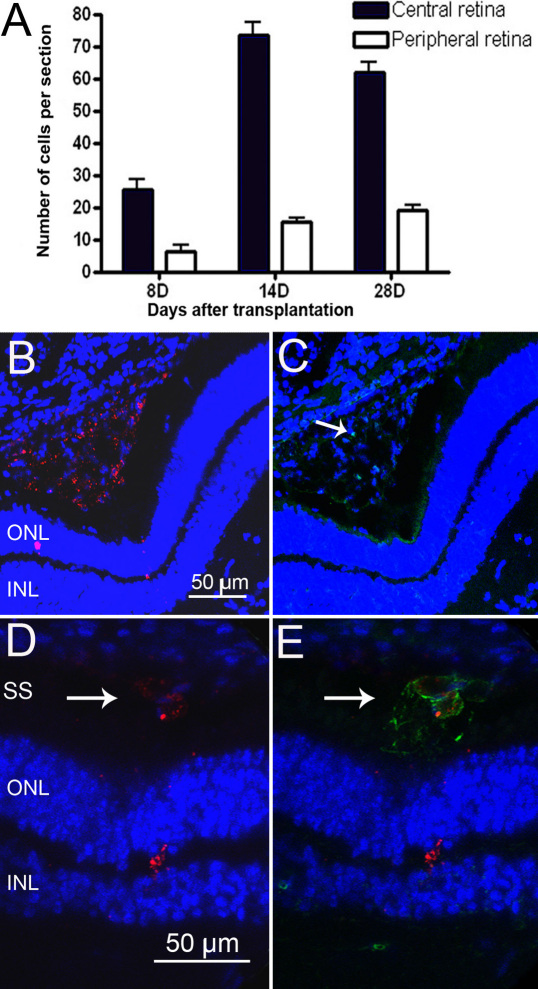
Analysis of migration, proliferation, and death of porcine ciliary epithelium (CE)-derived cells after subretinal transplantation. **A**: Quantification of the transplanted cells that had migrated into the neuroretina. CM-DiI-labeled cells were counted in 20 random sections from each eye. The middle third, containing the optic nerve, was considered to be the central retina and the two peripheral thirds, including the ora serrata, were considered to be the peripheral retina. The results are presented as the mean±SEM **B**, **C**: Cell proliferation assessed by Ki67 labeling in transplanted retinas. CM-DiI-positive cell aggregates in the subretinal space (red in **B**) contained rare Ki67-labeled cells (green, arrow in **C**), eight days after transplantation. **D**, **E**: Phagocytosis of transplanted cells by macrophages. CM-DiI-labeled particles (red in **D**, arrow) contained within isolectin B4-positive macrophages (green in **E**, arrow). The nuclei are labeled with 4',6-diamidino-2-phenylindole (DAPI; blue). Outer nuclear layer (ONL); inner nuclear layer (INL); and subretinal space (SS).

Some transplanted cells migrated into the neuroretina and both CM-DiI positive pigmented and non-pigmented cells were observed interspersed with host retinal cells. The number of CM-DiI-labeled cells in the central and peripheral neurororetina was quantified by counting the cells in transverse sections containing the optic nerve head. At all time points, a proportion of surviving CM-DiI-labeled cells was found in the peripheral retina, indicating that transplanted cells had migrated tangentially from the site of injection in the central retina to more peripheral sites (Figure 6A). Cell proliferation of the transplanted cells was assessed by immunolabeling for the cell proliferation marker Ki67. A small number of imunopositive cells were found within the CM-DiI-labeled cell aggregates in the subretinal space, but no Ki67 staining was observed in the neuroretina (Figure 6B,C). Therefore, the increase in CM-DiI cell numbers in the neuroretina—from 8 to 14 and 28 days—post transplantation is likely to be due to cell migration rather than proliferation of the transplanted cells within the retina. Isolectin B4 labeling was performed to identify the distribution of immune cells (macrophage/microglia) in the injected retinas. Round, large cells positive for isolectin were identified in the subretinal space. These cells contained red particles, suggesting phagocytosis of transplanted cells by macrophages (Figure 6D,E). However, cells double-labeled with CM-DiI and isolectin were not found within the neuroretina.

**Figure 7:**
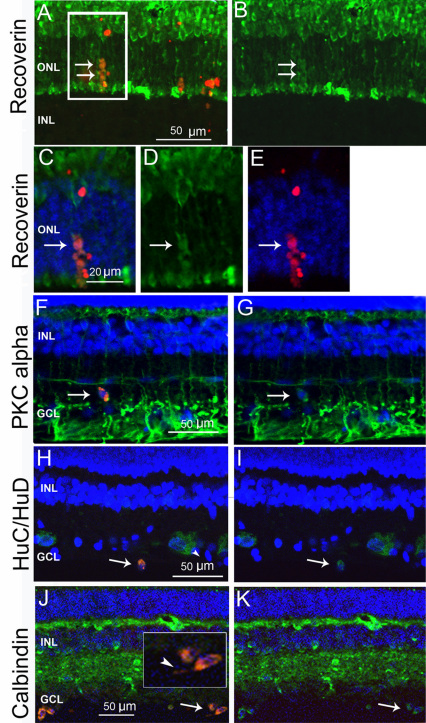
The immunoreactivity of transplanted ciliary epithelium (CE)-derived cells in the neuroretina. **A**: Microphotograph (13.3 µm confocal stack) showing red CM-DiI-labeled cells positive for recoverin (green, arrows) in the outer nuclear layer (ONL). **B**: Recoverin-only labeling of the same confocal stack as in **A**. The arrows point to the transplanted cells. **C**: A 0.7 µm confocal slice from the boxed area in **A,** showing double-labeled CM-DiI/recoverin positive cells (arrows) **D**: Green recoverin labeling in the same area (arrow). **E**: Red CM-DiI labeling and 4',6-diamidino-2-phenylindole (DAPI) nuclear staining of the same area. **F**, **G**: CM-DiI-labeled, protein kinase α (PKCα)-positive cell (green) in the inner plexiform layer (arrows). **H**, **I**: HuC/D (green) and CM-DiI-positive cell in the ganglion cell layer (GCL; arrow in **H** and **I**). **J**, **K**: Calbindin (green) and CM-DiI-labeled cells in the GCL (arrows). The inset is at a higher magnification with visible processes (arrowhead). The nuclei are labeled with DAPI (blue). Inner nuclear layer (INL).

To assess whether transplanted CE-derived cells that had migrated into the neuroretina displayed features suggestive of neuronal differentiation, sections of the transplanted eyes were assayed with antibodies for retinal cell markers. CM-DiI cells positive for the photoreceptor marker recoverin were detected in the ONL (Figure 7A-E). Double CM-DiI/PKCα-positive cells displayed oval shapes and were usually found outside the inner nuclear layer (INL), adjacent to bipolar cells (Figure 7F,G). PKCα labeling in pigs and cows is more intense in the ganglion cell layer (GCL), where it has been colocalized with astrocytes [18,25]. CM-DiI cells positive for the early neuronal marker HuC/D (Figure 7H,I) and calbindin-immunopositive cells were found in the GCL (Figure 7J,K), where they had a rounded or oval shape, with short thin processes (Figure 7J, inset). Since those cells were often observed close to the vitreal side, it cannot be excluded that they were retracted to the vitreous after the injection procedure, or had migrated back to the vitreous through the needle track. The percentage of CM-DiI-labeled cells localized in the neuroretina and positive for retinal neuronal markers was at an average of 8%–10% for recoverin, 5%–6% for PKCα, and 6%–9% for calbindin. The CM-DiI-labeled cells in the neuroretina always appeared slightly displaced from the pattern of the host retina and their morphology remained distinguishable from the recipients’ cells.

## Discussion

Recent reports have highlighted the limited understanding we have of the nature and developmental potential of CE-derived cells and the need for further investigations to re-evaluate their biology and potential for cell therapies in retinal degenerative diseases.

Several sources of cells have been considered for retinal cell replacement therapies and tested for their ability to generate retinal cell types. Transplanted retinal progenitors have been shown to express retinal cell markers in mice and pigs [21,26]. Functional cell replacement was demonstrated for the first time in a study by MacLaren et al. [27], where dissociated photoreceptor precursors from postnatal mice were used for transplantation. However, this strategy would be inadequate in humans, where the cells of the comparable ontogenic stage would have to be obtained from fetal retina, and being postmitotic, could not be expanded.

Human embryonic (hES) stem cells and induced pluripotent stem (iPS) cells are very attractive sources of cells for cell replacement. Efficient protocols for retinal differentiation of ES cells have been developed and are constantly improving [28-31]. The feasibility of using ES-derived photoreceptors has been demonstrated after their transplantation into adult Crx^−/−^ mice with subsequent improvement in visual function [32]. Similarly, photoreceptors have been generated from iPS cells and their integration into both mice and pig retina has been reported [33,34]. Recently, transplantation of iPS-derived photoreceptor precursors from mice has been shown to restore visual function in rho^−/−^ mice [35]. RPE cells have also been generated from hESC [36-40] and their transplantation rescued visual function in a rat model of retinal degeneration [41,42]. Cells with RPE features have also been differentiated from human iPSCs and they were able to delay retinal degeneration in animal transplantation studies [37,43]. Although the use of ES and iPS for cell replacement therapies shows great promise, issues such as oncogenic potential and immunogenicity have to be fully addressed before ES or iPS cells can be considered for treatment. Transplantation strategies for retinal replacement also require optimization due to the low rate of cell survival and the integration of transplanted cells. Efforts have been made to improve cell survival by transplanting cells on biodegradable scaffolds [44-47]. It has been identified that the outer limiting membrane (OLM) represents a barrier to cell integration and several approaches to controlling OLM disruption have led to enhanced integration of transplanted cells [48-52]. Until a successful method and a reliable cell-type for cell-based therapies in the retina have been identified, it is preferable to continue the investigation of cells from different sources and developmental origins with the potential to generate the differentiated progeny of interest. Although the differentiation potential of CE-derived cells is currently debatable, literature from the past decade suggests that the behavior of these cells in differentiation cultures is dependent on the experimental conditions. Such conditions could either promote a direct transition to the epithelial RPE-like phenotype or the development of retinal neuronal phenotypes, possibly through transdifferentiation or via de-differentiation and a stem-like transition state [53].

To gain insight into the differentiation potential of postnatal porcine CE-derived cells, we determined the expression of the key pluripotency genes in P1 spheres [23,24]. We were able to detect mRNAs for *Klf4*, *Sox2*, and *cMyc*, while the transcripts for *Nanog* and *Oct4* were absent. Cells positive for *cMyc*, but negative for *nMyc* were recently identified as a retinal stem cell population in *Xenopus* and in zebrafish ciliary margins. Therefore, it has been suggested that the expression patterns of *cMyc* and *nMyc* could be used to localize stem cells in the mammalian developing retina and CE [54]. The lack of expression of the whole set of pluripotentcy genes highlights the important differences between CE-derived cells and embryonic or iPS cells. Our PCR data are in agreement with recently published analysis of NRL-eGFP mice CE-derived cultures that could not be differentiated into photoreceptors when subjected to the retinal differentiation protocol for ES cells [12]. However, porcine CE-derived cells contained mRNAs for genes expressed during retinal development, from the optic vesicle stage (*Six3* and *Mitf*) to retinal histogenesis (*Hes1*, *Chx10*, and *Otx2*). Mitf transcription factor also plays a role in promoting and maintaining the RPE [55].

Our previous study has shown that the proliferation capacity of porcine CE-derived cells decreases with the age of the donor animal [8], suggesting that the cells from younger animals may be more stem-like, with a higher ability for retinal differentiation. However, it is important to note that retinal histogenesis is complete after birth, with all retinal layers and cell types present in newborn pigs [18]. It remains to be evaluated whether the capacity of porcine CE-derived cells to generate retinal cell types actually decreases with the age of the cell donor.

Cells from the first passage in our current study generated a higher number of photoreceptor-like cells compared to our previous study, where cells were used for differentiation after passage three [8]. The fact that cells from earlier passages possess higher differentiation potential is a limiting factor for the expansion of CE-derived cells. This issue requires our attention and it will have to be resolved if these cells are to be used for cell replacement. In our current study, colabeling for rhodopsin and recoverin and the lack of cells double-labeled for recoverin and PKCα confirmed the photoreceptor-like phenotype of the differentiated cells. Interestingly, PKCα-labeled cells were always found to be closely associated with recoverin-positive cells, suggesting that they might influence each other’s differentiation.

Expression of neuronal cell markers in our differentiation cultures coincided with significant, though not always complete, loss of pigmentation. Persistence of some pigmented granules in differentiated cells indicates that they originate from pigmented cells, but may require additional time to clear their pigment content.

The differentiation potential of porcine CE-derived cells in vitro was also previously studied by MacNeil et al. [19]. Although the expression of generic neuronal markers such as β-III-Tubulin and Neu-N was demonstrated, no expression of more specific retinal markers was detected in differentiation cultures in their study. The discrepancy relative to our results could be explained by the difference in the donor animal’s age, the post-mortem time before cell isolation, or by the conditions for in vitro differentiation. Retention of growth factors in our differentiation cultures increased the number of differentiated cells, suggesting a role for growth factors in the differentiation and/or survival of CE-derived cells. Notably, EGF does not affect in vitro photoreceptor survival in rats [56], but it can stimulate the survival of porcine photoreceptors under the same conditions [57]. In addition to the role of both bFGF and EGF in neuronal and retinal differentiation [58-61], bFGF has been shown to play a role in transdifferentiation of RPE and iris pigment epithelium into retinal tissue [62-64]. Finally, although EGF was reported to be a negative regulator of photoreceptor differentiation during retinal development [65,66], it has been shown, in vitro, to act as a neuronal differentiation factor for retinal stem cells [67].

In vivo, transplanted CE-derived cells showed remarkable migration potential as they were found in the peripheral regions of the RPE and retina. The injection of dissociated cells rather than of intact spheres, as performed in other studies, might have facilitated migration [11].

We identified the recruitment of isolectin B-positive macrophages to the subretinal space of injected eyes with the presence of red particles contained within the phagocytes. Given the relatively short timeframe, an active immune rejection of subretinal allografts is unlikely to have been induced [21,27], therefore the macrophages are probably responsible for scavenging cell debris from the dead transplanted cell.

Some transplanted CE-derived cells that had migrated into the neuroretina displayed positive and specific immunostaining for neuronal cell markers. The markers shown to be expressed in vitro in our current and previous study [8] were also detected in CE-derived cells after transplantation. All antibodies used in this study have been carefully and extensively characterized on pig tissue [18], therefore, nonspecific antibody labeling of transplanted cells is unlikely. Furthermore, the CM-DiI dye was previously successfully used for the long-term follow-up of neural stem cells after transplantation, and no diffusion of the dye was reported [68].

The majority of transplanted cells formed multilayered RPE-like structures positive for RPE65. Although, in vivo we observed preferential differentiation of CE-derived cells along the RPE lineage, immunoreactivity for the RPE marker RPE65 in vitro was relatively low, suggesting that the differentiation protocol we adopted was not optimal for the efficient generation of RPE-like phenotypes. Indeed, our protocol was designed to generate retinal neurons. However, the culture conditions for efficient in vitro differentiation of CE-derived cells into the RPE phenotype have recently been reported [69]. Cells generated with this protocol had epithelilal morphology, immunocytochemical, and ultrastructural features of RPE and a capacity for phagocytosis. Another study has shown that high expression of RPE65 can be induced in CE-derived cells in a medium supplemented with vasoactive intestinal peptide (VIP) or in a RPE cell-conditioned medium [70].

In vivo, differentiation along both the RPE and neuronal lineages could be advantageous. Cell therapies can be most effective if the contribution of different cell types is harnessed, not only to replace lost cells, but also to maintain existing function and prevent further degeneration. CE-derived RPE-like cells could contribute a protective effect by promoting photoreceptor survival [37], while newly differentiated photoreceptors could replace lost ones. Indeed, subretinal transplantation of sheets of human retinal progenitor cells together with their RPE is the only method thus far shown to be effective in humans [46]. Promising results in generating functional photoreceptors from CE-derived cells in vitro and in vivo after gene transfer and modulation of transcription factors have recently been reported [15,16].

In conclusion, our study shows that the cells from postnatal pig CEs have the ability to generate cells with the morphological and immunohistochemical features of retinal neurons and RPE, both in vitro and after subretinal transplantation in vivo. Revealing the paracrine effects and the influence of the cellular environment in determining the fate of these cells may identify specific factors that enable controlled differentiation, or in vivo activation of these cells. Understanding the pathways behind this cell plasticity may provide important clues for the development of future cell replacement therapies to combat retinal degeneration.
